# Optimal parity cut-off values for predicting postpartum hemorrhage in vaginal deliveries and cesarean sections

**DOI:** 10.11604/pamj.2020.37.336.24065

**Published:** 2020-12-11

**Authors:** Yasuhiro Miyoshi, Sanford Khondowe

**Affiliations:** 1 Zimba Mission Hospital, Zimba, Zambia

**Keywords:** Zambia, postpartum hemorrhage, risk factor, high parity, cut-off value

## Abstract

**Introduction:**

high parity is a major public health concern in developing countries and it is a risk factor for postpartum hemorrhage (PPH). The aim of this study was to analyze the optimal parity cut-off values for predicting PPH in vaginal deliveries and cesarean sections in a rural Zambian setting.

**Methods:**

all women who delivered at Zimba Mission Hospital in 2017 were reviewed in this retrospective survey. Those whose records were missing data on parity and those with risk factors for developing PPH (e.g. birth weight ≥4,000 g, multiple pregnancy, assisted vaginal delivery and placenta previa) were excluded. We analyzed the association between parity and PPH using multiple logistic regression and ROC curve analyses.

**Results:**

among the 1,555 women included in the study, 72 (4.6%) women experienced PPH. The optimal cut-off values for parity in vaginal deliveries and cesarean sections were para 7 and 3, respectively. Using these cut-off values, the adjusted odds ratios (95% confidence intervals) were 3.26 (1.15, 9.21) and 8.28 (2.25, 30.5), respectively.

**Conclusion:**

proper preparation is required for vaginal deliveries in women with a history of ≥7 births and cesarean sections in women with a history of ≥3 births.

## Introduction

Postpartum hemorrhage (PPH) is the leading cause of maternal mortality, accounting for 27.1% of all maternal deaths [1]. Although maternal mortality rates have declined greatly in developed countries, PPH remains a serious problem in developing countries, where more than 99% of maternal mortality due to PPH occurs [1]. Thirty-four percent of maternal mortality in Zambia is attributable to PPH [2].

An understanding of risk factors is important to properly prepare for deliveries. High parity is a risk factor for PPH [3-5]. The prevalence of grand multi-parity (para 5 and above) [6] is as low as 3-4% in developed countries, while it is 19.3% in developing countries [7-9]. Associated factors include a high rate of unmet contraceptive needs and low socio-economic status [10]. The national referral guideline in Zambia states that grand multiparous women should delivery in hospitals rather than health centers [11].

However, what qualifies as high parity and whether para 5 is the optimal cut-off value remain to be elucidated. Additionally, no studies have investigated whether the cut-off value changes according to the mode of delivery. The aim of this study was to analyze the optimal parity cut-off values for predicting PPH in vaginal deliveries and cesarean sections in a rural Zambian setting.

## Methods

**Study design, population and setting:** this is a secondary analysis on data from a previously published study on PPH [12]. This retrospective cohort study was conducted at Zimba Mission Hospital in southern province, Zambia. This district hospital is located 400 km south of Lusaka, the capital of Zambia. It accepts patients referred from as many as 10 health centers in the catchment area, which has a population of 98,000. The study population included all women who delivered at the hospital between January 1^st^ and December 31^st^, 2017. Those whose records were missing data on parity and those with previously reported risk factors for developing PPH [13] (e.g. birth weight ≥4,000 g, multiple pregnancy, assisted vaginal delivery (by vacuum or forceps)) and placenta previa were excluded to figure out the independent effect of parity on PPH.

**Data collection and definitions:** demographic, clinical and outcome data were extracted from the admission, delivery and operation registers for all patients during the study period. Blood loss was estimated after delivery by the attending clinician (a midwife or doctor). PPH was defined as ≥500 ml blood loss in the 24 h after vaginal delivery or ≥1,000 ml blood loss in the 24 h after cesarean section [13]. Active management of the third stage of labor (AMTSL), consisting of the intramuscular injection of oxytocin (10 IU), controlled cord traction and uterine massage, was performed for all patients who delivered vaginally. Additional oxytocin was given, and bimanual uterine compression was performed in cases of PPH. Patients undergoing cesarean section also received oxytocin (10 IU). The management of the third stage of labor and PPH were based on the guideline proposed by University Teaching Hospital, Lusaka, Zambia [14].

**Statistical analysis:** data were entered in Microsoft excel (version 14.1.0; Microsoft®, Redmond, WA) and exported to EZR (version 3.1.2; Saitama Medical Center, Jichi Medical University, Saitama, Japan), which was used to perform the statistical analysis. The Mann Whitney U test was used to analyze continuous variables and Pearson's chi-squared test was used to analyze qualitative variables. A multiple logistic regression analysis was performed to identify independent variables. Factors with p values <0.05 according to the Mann Whitney U test or Pearson's chi-squared test were entered into the multivariate analysis. P values of <0.05 were considered to indicate statistical significance. The optimal parity cut-off values for predicting PPH in vaginal deliveries and cesarean sections were analyzed by multiple logistic regression and ROC curve analyses.

**Ethics approval and consent to participate:** this study was approved by the research ethics committees of the University of Zambia Biomedical Research Ethics Committee (No. 001-03-19). The hospital administrator at Zimba Mission Hospital has granted permission to conduct the research after the ethical approval was obtained at an executive meeting. No ethical issue arose during this study, as it was retrospective and all data were anonymous.

## Results

**Figure 1:**
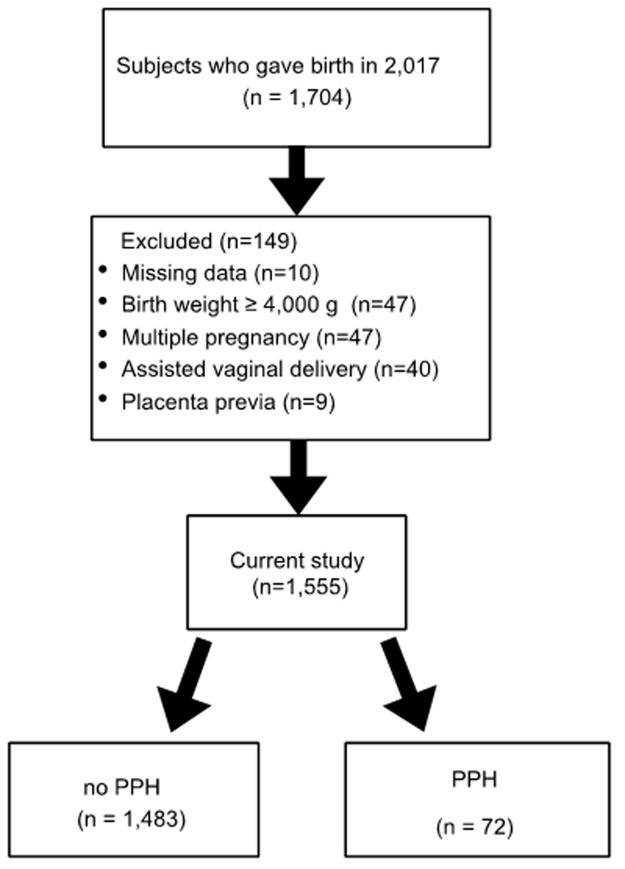
flow chart of the patients included in this study

A total of 1,704 women were reviewed and 1,555 women were included in the current study after excluding 149 cases for the following reasons: missing data on parity (n=10), birth weight ≥4,000 g (n=47), multiple pregnancy (n=47), assisted vaginal delivery (n=40) and placenta previa (n=9) (Figure 1). The maternal and neonatal characteristics are listed in Table 1. Women with PPH tended to be older (p=0.002) and have higher parity (p<0.001). The rate of previous cesarean section (p=0.017) and the cesarean section at the current pregnancy (p<0.001) was higher in the PPH group. Birth weights tended to be greater in the PPH group (p=0.010) (Table 1). The multiple logistic regression analysis demonstrated that cesarean section at the current pregnancy was the only factor significantly associated with PPH (p<0.001) and the association between parity as a continuous variable and PPH lost significance after adjusting for confounders (Table 2).

**Table 1:** maternal and neonatal characteristics

Characteristic	No PPH (n=1,483)	PPH (n=72)	p
Maternal age (years)*	24 (19-33)	30 (22-36)	0.002
Parity*	1 (0-4)	3 (1-6)	<0.001
Previous cesarean section*	64 (4.3)	8 (11.1)	0.017
HIV seropositivity	73 (4.9)	4 (5.6)	>0.999
HDP	32 (2.2)	3 (4.2)	0.474
Gestational age of delivery (weeks)	39 (37-40)	39 (38-40)	0.876
Cesarean section*	231 (15.6)	30 (41.7)	<0.001
Birth weight (kg)*	3.05 ± 0.47	3.14 ± 0.58	0.010

Values are shown as the median (25^th^-75^th^ percentile) or number (percentage); PPH, postpartum hemorrhage; HIV, human immunodeficiency virus; HDP, hypertensive disorders of pregnancy; *p<0.05

**Table 2:** multiple logistic regression analysis for PPH

Characteristic	Adjusted OR (95% CI)	p
Maternal age (years)	1.01 (0.96, 1.07)	0.697
Parity	1.13 (0.96, 1.34)	0.139
Previous cesarean section	1.26 (0.54, 2.97)	0.595
HIV seropositivity	1.02 (0.36, 2.95)	0.966
HDP	1.90 (0.55, 6.57)	0.311
Gestational age of delivery (weeks)	0.96 (0.86, 1.06)	0.378
Cesarean section*	3.83 (2.22, 6.61)	<0.001
Birth weight (kg)	1.12 (0.64, 1.93)	0.697

PPH, postpartum hemorrhage; HIV, human immunodeficiency virus; HDP, hypertensive disorders of pregnancy; OR, odds ration; CI, confidence interval; *p<0.05

To investigate the optimal parity cut-off values for the prediction of subsequent PPH, we analyzed the association between parity and PPH separately in vaginal deliveries and cesarean sections. The incidence of PPH and the relative odds ratios (OR) for developing PPH were calculated (Table 3, Table 4). The statistical analysis revealed that, in vaginal deliveries, para ≥7 was associated with a significantly greater risk of PPH (Odds ratio 3.26; 95% confidence interval (95% CI) 1.15, 9.21; p=0.025). For para ≥7, the positive predictive value (PPV), negative predictive value (NPV), sensitivity and specificity were 7.5%, 97.2%, 19.0% and 92.3% respectively.

**Table 3:** incidences and odds ratios for PPH in vaginal deliveries in subgroups divided by different number of parity

Parity	Incidence of PPH (below vs above)	Adjusted OR (95% CI)	p
1	3.5% (15/428) vs. 3.1% (27/866)	0.48 (0.19-1.23)	0.127
2	3.5% (22/627) vs. 3.0% (20/667)	0.38 (0.14-1.02)	0.055
3	2.9% (22/767) vs. 3.8% (20/527)	1.23 (0.45-3.36)	0.685
4	2.7% (24/876) vs. 4.3% (18/418)	2.05 (0.73-5.74)	0.172
5	2.9% (28/975) vs. 4.4% (14/319)	1.70 (0.62-4.65)	0.302
6	2.9% (32/1,085) vs. 4.8% (10/209)	1.69 (0.62-4.61)	0.305
7*	2.9% (34/1,189) vs. 7.6% (8/105)	3.26 (1.15-9.21)	0.025
8	3.2% (40/1,244) vs. 4.0% (2/50)	1.08 (0.23-5.04)	0.927
9	3.2% (41/1,278) vs. 6.2% (1/16)	1.77 (0.21-14.80)	0.598
10	3.5% (42/1,286) vs. 0% (0/8)	NA	NA

**Table 4:** incidences and odds ratios for PPH in cesarean sections in subgroups divided by different number of parity

Parity	Incidence of PPH (below vs. above)	Adjusted OR (95% CI)	p
1*	0.9% (1/112) vs. 19.5% (29/149)	28.8 (3.02-275)	0.004
2*	2.6% (4/153) vs. 24.1% (26/108)	10.0 (2.38-42.2)	0.002
3*	3.9% (7/179) vs. 28.0% (23/82)	8.28 (2.25-30.5)	0.001
4	7.2% (14/194) vs. 23.9% (16/67)	1.18 (0.36-3.90)	0.784
5	7.8% (16/205) vs. 25.0% (14/56)	1.06 (0.34-3.34)	0.921
6	9.4% (21/223) vs. 23.7% (9/38)	0.74 (0.24-2.24)	0.592
7	10.1% (24/238) vs. 26.1% (6/23)	0.76 (0.22-2.62)	0.660
8	10.9% (27/248) vs. 23.1% (3/13)	0.41 (0.07-2.33)	0.312
9	11.7% (30/256) vs. 0% (0/5)	NA	NA
10	11.6% (30/259) vs. 0% (0/2)	NA	NA

PPH: postpartum hemorrhage; OR: odds ration; CI: confidence interval; NA: not applicable; *p<0.05

**Figure 2:**
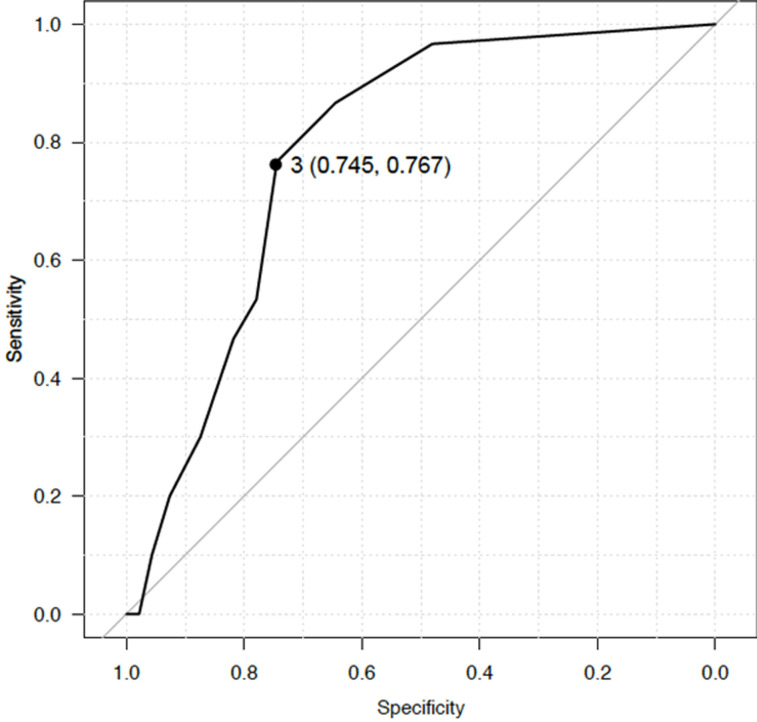
the ROC curve analysis for optimal cut-off value for predicting PPH in cesarean sections

In cesarean sections, parity cut-off values of 1-3 were significantly associated with PPH. The ROC curve analysis showed that para 3 was the optimal cut-off value for predicting PPH with area under the ROC curve (AUC) of 0.787 (Figure 2). Using para 3 as the cut-off value (OR 9.58; 95% CI 3.91, 23.5; p<0.001), the PPV, NPV, sensitivity and specificity were 28.1%, 96.1%, 76.7% and 74.5% respectively. All 4 cases of uterine rupture in patients without a history of cesarean section or uterine operation occurred in women with a history of ≥3 births (para 3, para 5, para 5 and para 6 respectively). All of these women were referred from health centers during labor and cesarean section was performed at the hospital.

## Discussion

This study demonstrated that optimal parity cut-off values for predicting PPH in vaginal and cesarean section deliveries were para 7 and para 3 respectively. To the best of our knowledge, this is the first study to demonstrate the optimal parity cut-off values for indicating the risk of PPH according to the mode of delivery. Previous studies [3-5] have shown an association between high parity and PPH. However, these studies did not demonstrate the optimal cut-off values. Some previous studies showed that grand multi-parity was not associated with PPH [7,10]. However, this might be because para 5 is not the optimal cut-off.

As parity increases, a woman´s myometrial muscular strength may decrease due to a reduction of collagen fibers [5]. Therefore, when parity increases, the probability of experiencing PPH increases. The discordancy of parity cut-off values between vaginal delivery and cesarean section indicates that cesarean section might affect the function of collagen fibers. The association between parity as continuous variable and PPH lost significance after adjustment for confounders. However, parity as a categorical valuable, when using the specified cut-off value, was significantly associated with PPH. This suggests that the association between parity and PPH is not completely linear. Our result is consistent with a previous study in Australia [15].

The study findings suggest that healthcare workers should be well prepared to deal with the women with high parity. Several health professionals should attend vaginal delivery for women with a history of ≥7 births. Sufficient blood for transfusion should be prepared before performing cesarean section for the women with a history of ≥3 births because more blood loss is expected in comparison to vaginal delivery [12].

The present study was associated with some limitations. First, higher parity women were small in number. Thus, it might skew the analysis. Second, the diagnosis of PPH was based on estimated (rather than measured) blood loss. Third, the gestational age data may not have been accurate because ultrasound is usually unavailable during early pregnancy and expected due dates are determined based on the last menstrual period, which is subject to memory bias. Fourth, the registers did not contain data on the previous history of PPH or the body mass index, which prevented the evaluation of the effects of these known risk factors for PPH in the present study [16,17]. Fifth, the data were collected at one district hospital. Thus, it might be difficult to generalize these data to the whole population of Zambia or other countries.

## Conclusion

A history of ≥7 births in vaginal deliveries and ≥3 births in cesarean section deliveries was associated with PPH. During labor, providers caring for patients with these risk factors should be prepared to manage PPH.

### What is known about this topic

- High parity is a risk factor for PPH;
- The prevalence of grand multiparity is much higher in developing countries than in developed countries.

### What this study adds

- The optimal parity cut-off value for predicting PPH in vaginal deliveries was para 7;
- The optimal parity cut-off value for predicting PPH in cesarean section deliveries was para 3.
